# Supplementary figures and images for: Dynamical Model of Drug Accumulation in Bacteria: Sensitivity Analysis and Experimentally Testable Predictions
Source: PLoS One. 2016 Nov 8;11(11):e0165899. doi: 10.1371/journal.pone.0165899 (PMC5100933; doi:10.1371/journal.pone.0165899)

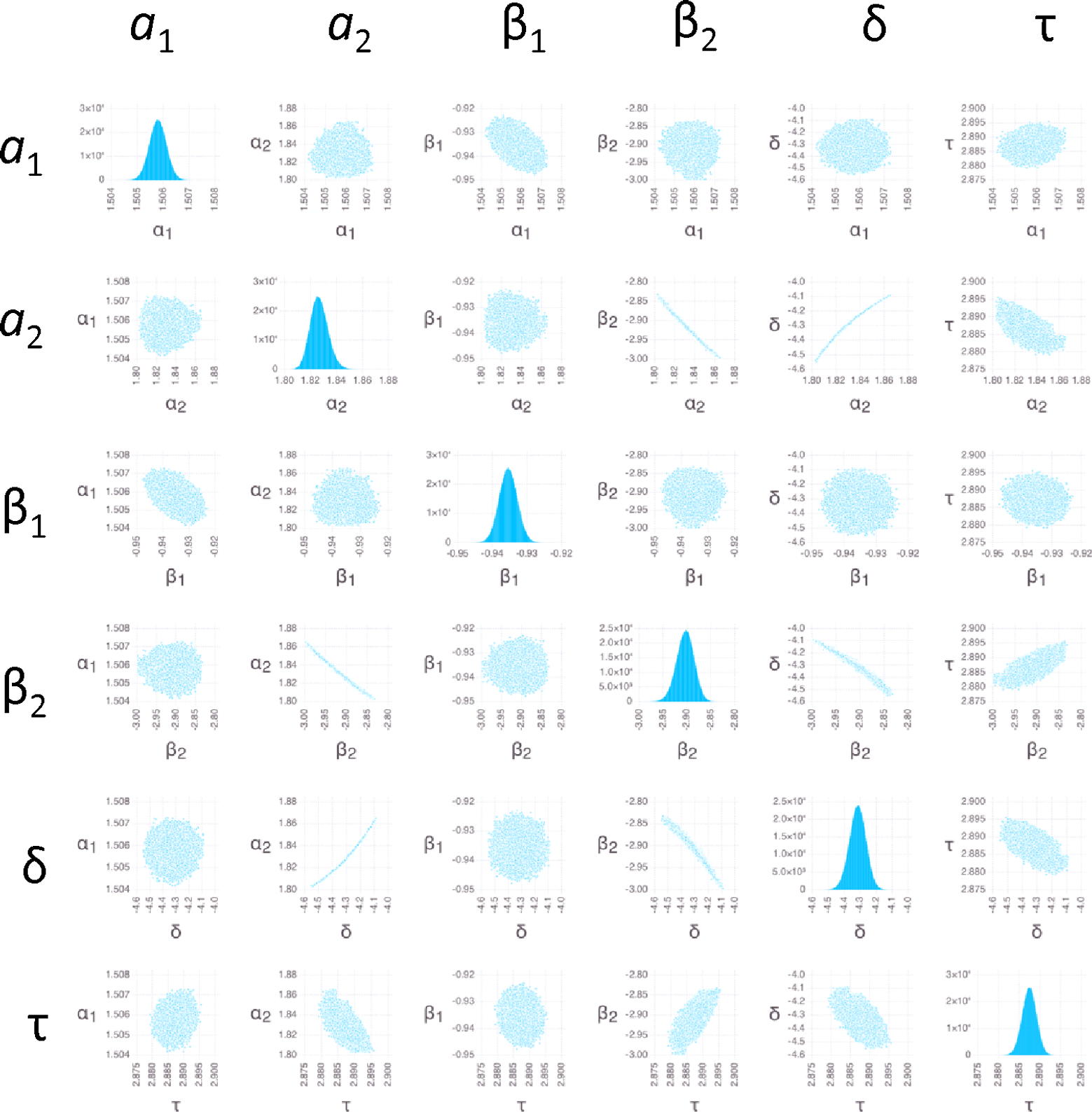

Supplement: S1 Fig — The analysis and presentation is the same as in Fig 5, but using a 10-fold lower error value. (TIF) [file pone.0165899.s001.tif]

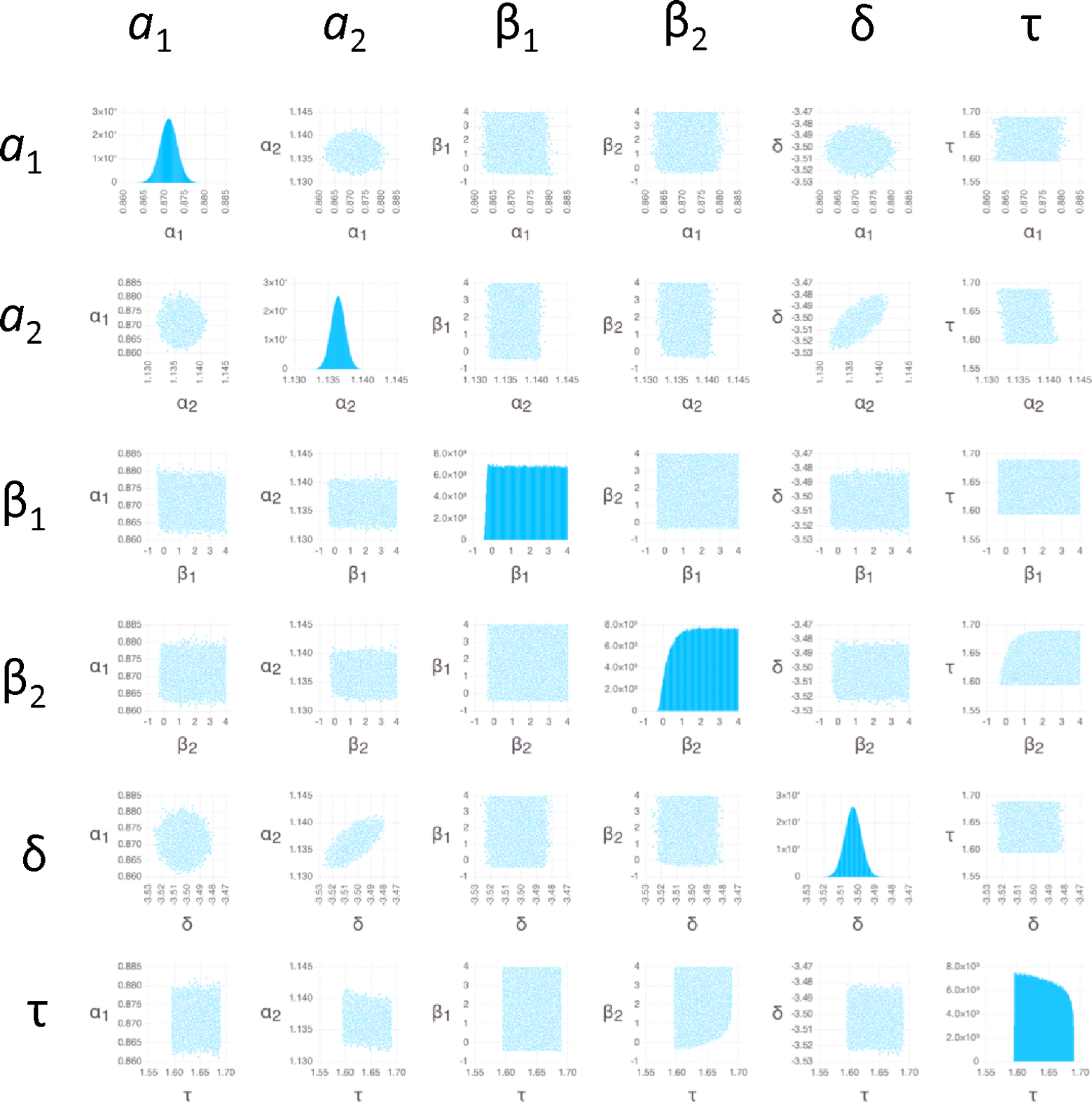

Supplement: S2 Fig — The analysis and presentation is the same as in Fig 6, but using a 10-fold lower error value. (TIF) [file pone.0165899.s002.tif]

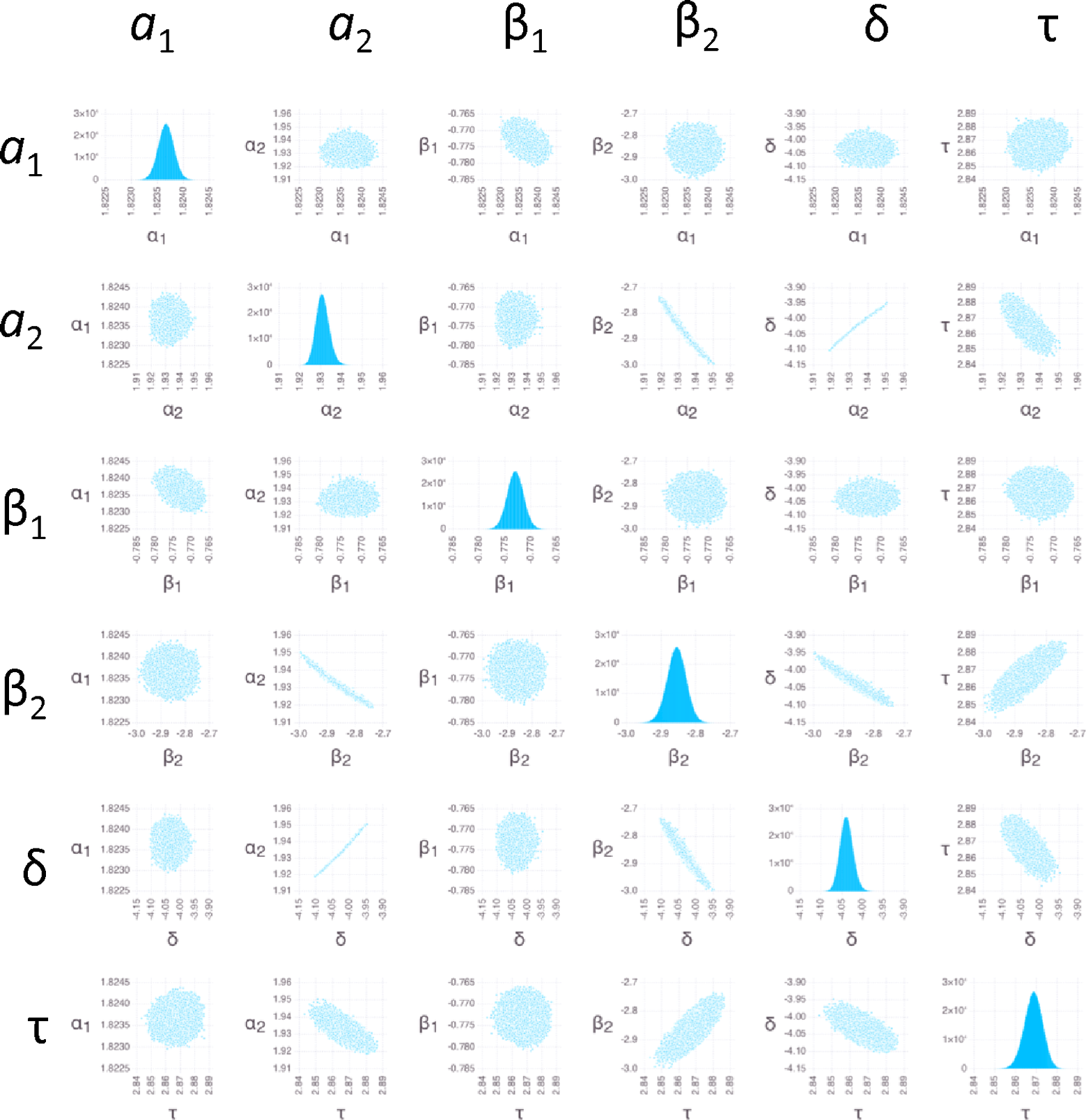

Supplement: S3 Fig — The analysis and presentation is the same as in Fig 7, but using a 10-fold lower error value. (TIF) [file pone.0165899.s003.tif]
