# Supplementary material for: Dynamical Model of Drug Accumulation in Bacteria: Sensitivity Analysis and Experimentally Testable Predictions
Source: PLoS One. 2016 Nov 8;11(11):e0165899. doi: 10.1371/journal.pone.0165899 (PMC5100933; doi:10.1371/journal.pone.0165899)
Supplement: S1 Table — (DOCX) [file pone.0165899.s005.docx]

Table S1. Parameter rankings based on different sensitivity methods (values in bold are representing ranking ties).

| Method | *a*_1_ | *a*_2_ | β_1_ | β_2_ | δ | τ |
| --- | --- | --- | --- | --- | --- | --- |
| *E. coli* | | | | | | |
| GSA | 3 | 1 | 4 | 5 | 6 | 2 |
| LSA | 2 | 1 | 5 | 4 | 6 | 3 |
| BSA | 1 | 2 | 4 | 5 | 6 | 3 |
| *P. aeruginosa* | | | | | | |
| GSA | 2 | 1 | ***5*** | ***5*** | 4 | 3 |
| LSA | 3 | 1 | 4 | 6 | 2 | 5 |
| BSA | 3 | 1 | ***5*** | ***5*** | 4 | 2 |
| *S. aureus* | | | | | | |
| GSA | ***3*** | 1 | ***3*** | 5 | 6 | 2 |
| LSA | 1 | 2 | 6 | 4 | 3 | 5 |
| BSA | ***1*** | ***1*** | 4 | 5 | 6 | 3 |
